# Breast cancer resistance to chemotherapy: When should we suspect it and how can we prevent it?

**DOI:** 10.1016/j.amsu.2021.102793

**Published:** 2021-09-04

**Authors:** Prihantono, Muhammad Faruk

**Affiliations:** Department of Surgery, Faculty of Medicine, Hasanuddin University, Makassar, Indonesia

**Keywords:** Breast cancer, Chemotherapy, Chemoresistance, Biomarkers, Assays

## Abstract

Chemotherapy is an essential treatment for breast cancer, inducing cancer cell death. However, chemoresistance is a problem that limits the effectiveness of chemotherapy. Many factors influence chemoresistance, including drug inactivation, changes in drug targets, overexpression of ABC transporters, epithelial-to-mesenchymal transitions, apoptotic dysregulation, and cancer stem cells. The effectiveness of chemotherapy can be assessed clinically and pathologically. Clinical response evaluation is based on physical examination or imaging (mammography, ultrasonography, computed tomography scan, or magnetic resonance imaging) and includes tumor size changes after chemotherapy. Pathological response evaluation is a method based on tumor residues in histopathological preparations. We should be suspicious of chemoresistance if there are no significant changes clinically according to the Response Evaluation Criteria in Solid Tumors and World Health Organization criteria or pathological changes according to the Miller and Payne criteria, especially after 2–3 cycles of chemotherapy treatments. Chemoresistance is mostly detected after the administration of chemotherapy drugs. No reliable parameters or biomarkers can predict chemotherapy responses appropriately and effectively. Well-known parameters such as cancer type, grade, subtype, estrogen receptor, progesterone receptor, human epidermal growth factor receptor 2, Ki-67, and MDR-1/P-gP have been used for selecting chemotherapy regimens. Some new methods for predicting chemoresistance include chemosensitivity and chemoresistance assays, multigene expressions, and positron emission tomography assays. The latest approaches are based on evaluation of molecular processes and the metabolic activity of cancer cells. Some methods for preventing chemoresistance include using the right regimen, using some combination of chemotherapy methods, conducting adequate monitoring, and using drugs that could prevent the emergence of multidrug resistance.

## Highlights

• Chemotherapy is an essential treatment in the management of breast cancer.
• Chemotherapy is carried out based on the selection of regimens for the specific individual and tumor characteristics.
• Combination therapy, monitoring, and evaluation are used to prevent chemoresistance.

## 1 Introduction

Breast cancer is the most commonly diagnosed cancers and the leading cause of death from cancer in women worldwide [1,2]. Data from the Jakarta Cancer Registry show that breast cancer has the highest incidence among cancers [3]. Most breast cancer patients in Indonesia present in advanced stages, with 63% in stages III and IV at diagnosis [4]. Based on data from Wahidin Sudirohusodo Hospital in Makassar in 2014, chemotherapy was performed on 247 breast cancer patients, neoadjuvant chemotherapy was performed on 145 (59%), and adjuvant chemotherapy was performed on 102 (41%). This resulted in a cumulative chemotherapy complete response rate of 2%, partial response rate of 78%, stable disease rate of 15%, and progressive disease rate of 5% [5].

Breast cancer is managed with multidisciplinary treatments including surgery, chemotherapy, radiation, hormonal therapy, and targeted therapy [6]. Chemotherapy is still a very important treatment in breast cancer [2,6]. Neoadjuvant chemotherapy has become standard in managing locally advanced breast cancer and is a treatment choice in early-stage operable breast cancer [7].

Chemotherapy can induce tumor cell death and reduce tumor mass, but some patients experience recurrence and death due to failure in treatment [8]. Chemoresistance is a problem that limits the effectiveness of chemotherapy [9]. It has two mechanisms: the tumor can be intrinsically resistant before administration of chemotherapy, and tumors previously sensitive to chemotherapy can show resistance during treatment [10].

The response to chemotherapy varies greatly. Some patients can achieve a complete response with just one or two cycles, while others require eight or more cycles [10]. This paper discusses data on chemotherapy responses, chemoresistance mechanisms, evaluation of chemotherapy responses, and predictors and prevention of chemotherapy responses in breast cancer. The review aimed to provide a better insight into the breast cancer response to chemotherapy.

## 2 Chemotherapy response

Chemotherapy improves outcomes in breast cancer patients, but the effect of cytotoxic treatment cannot be predicted for individual patients. Therefore, the identification of tumor characteristics associated with tumor response and outcomes is of great clinical interest. Several studies have been conducted to assess chemotherapy responses. Some clinical responses to neoadjuvant chemotherapy in previous studies are summarized in Table 1.

**Table 1:** Responses to neoadjuvant chemotherapy based on various studies.

Reference	n	Regimen	oCR	cPR
Fisher et al. [11]	760	AC	79	13
Amat et al. [11]	88	D	68	20
Bear et al. [11]	805 804	AC + D AC	91 86	26 13
Miller et al. [11]	40	A + D	85	10
Smith et al. [11]	52 52	ACV + D ACV	85 64	31 15
Von Minckwitz et al. [12]	441 444	AC + D A + D	85 75	14 7
Data from Wahidin Sudirohusodo Hospital [5]	145	CAF and TAC	80	N/A

Note: oCR, Overall clinical response (complete and partial); cPR, Complete pathological response; A, Adriamycin; AC, Adriamycin–cyclophosphamide; ACV, Adriamycin–cyclophosphamide–vincristine; D, Docetaxel; CAF, Cyclophosphamide–doxorubicin–5-fluorouracil; TAC, docetaxel–doxorubicin–cyclophosphamide.

Table 2 shows data on clinical responses to neoadjuvant chemotherapy in Wahidin Sudirohusodo Hospital [5].

**Table 2:** Cumulative clinical responses of breast cancer in Wahidin Sudirohusodo Hospital [5].

RECIST criteria	n (%)	%	Responses
CR	3 (2)	80	Responsive
PR	112 (78)		
SD	23 (15)	20	Nonresponsive
PD	9 (5)		

Note: CR, Complete response; PR, Partial response; SD, Stable disease; PD, Progressive disease.

Data from the hospital indicate a cumulative clinical complete response rate of 2%, partial response rate of 78%, stable disease rate of 15%, progressive disease rate of 5%, responsiveness rate of 80%, and nonresponsiveness rate of 20%. According to the data, the majority of the breast cancer patients who underwent chemotherapy had a partial response.

**Fig. 1:**
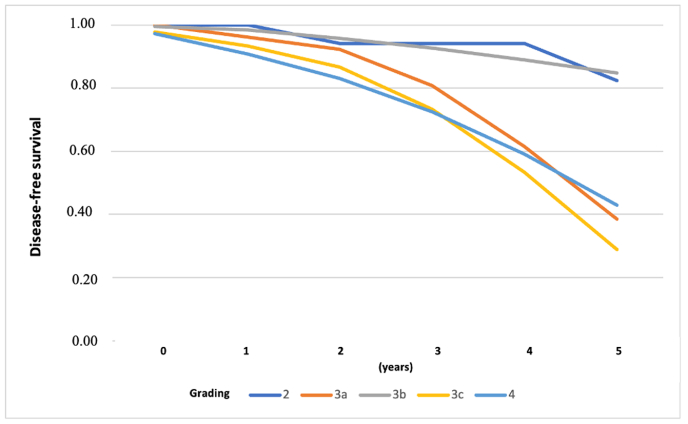
Curve of DFS based on severity grade in Wahidin Sudirohusodo Hospital [5].

The study results in Table 3 show the cumulative clinical responses based on subtype, such as a luminal rate of 66.67%, HER-2 rate of 70.37%, and triple-negative rate of 88.24%. The cumulative clinical response rates based on regimen were a cyclophosphamide–doxorubicin–5-fluorouracil (CAF) rate of 76.09%, docetaxel–doxorubicin (TA) rate of 87.50%, and docetaxel–doxorubicin–cyclophosphamide (TAC) rate of 72.41% [5]. A total of 161 cases (38.33%) were obtained; 259 cases were disease-free during the study period, with a disease-free survival (DFS) rate of 61.67% (Fig. 1).

**Table 3:** Clinical responses, subtypes, and regimens for breast cancer in Wahidin Sudirohusodo Hospital [5].

Factor	Number of patients	Chemotherapy response	Univariate analysis	
		cCR and PR (%)	OR (95% CI)	*p-*value
SUBTYPE				
Luminal	30	20 (66.67)	1.00	
Her2	27	19 (70.37)	1.19 (0.33–4.27)	0.763
Triple-negative	17	15 (88.240)	3.75 (0.63–39.2)	0.103
**REGIMEN**				
CAF/CEF	92	70 (76.09)	1.00	
TAC	29	21 (72.41)	0.825 (0.29–2.47)	0.69
TA/TE	17	14 (87.50)	2.2 (0.44–21.30)	0.31

Note: cCR, Clinical complete response; PR, Partial response; CAF, Cyclophosphamide–doxorubicin–5-fluorouracil; CEF, Cyclophosphamide–epirubicin–5-fluorouracil; TAC, Docetaxel–doxorubicin–cyclophosphamide; TA, Docetaxel–doxorubicin; TE, Docetaxel–epirubicin.

**Fig. 2:**
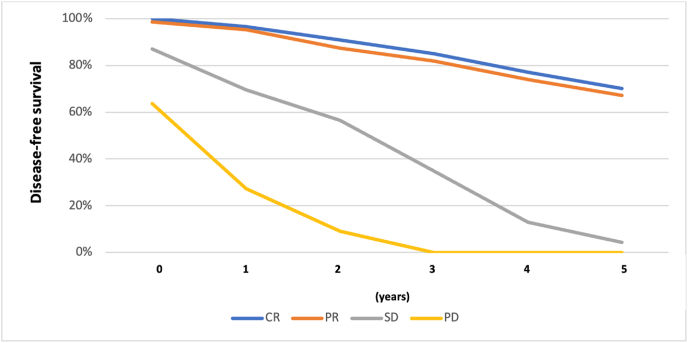
Curve of DFS based on chemotherapy responses in Wahidin Sudirohusodo Hospital [5].

Fig. 2 shows the differences between DFS (complete response and partial response) and non-responsiveness (stable disease and progressive disease) of cases.

## 3 Chemoresistance mechanism

Chemotherapy resistance is an obstacle to treating neoplasms and compromises the choice of chemotherapy in cases of recurrence. Therefore, choosing an accurate and effective treatment, in a more personalized way, for each patient is increasingly important.

Resistance limits the effectiveness of chemotherapy. Tumors could be intrinsically resistant before chemotherapy or during treatment, especially tumors that initially show sensitivity [2,13]. Therefore, understanding the mechanism of chemoresistance is very important to developing a cancer therapy approach [13]. The problems of drug resistance are complex. Many factors affect drug sensitivity, such as drug metabolism, activation and inactivation of drugs, elimination of chemotherapy drugs that are controlled by drug transporters and drug-metabolizing enzymes, changes in drug targets, DNA methylation, DNA repair, and avoidance of apoptosis [14].

One frequently investigated factor in chemotherapy resistance is the *MDR1* (multi-drug resistance) gene. The P-glycoprotein (P-gp) gene encodes a protein that acts as an efflux pump drug and removes drugs from cells, such as anthracyclines, etoposide, vinca alkaloids, dactinomycin, and paclitaxel [15]. Normally, P-gp in tissue cells has an excretion function, responsible for protecting cells from harmful chemicals in food. The ability of P-gp increases with overexpression of the *MDR1* gene, resulting in increased activity of drug efflux and resistance to chemotherapy [15,16]. This is related to research by Burger et al., who found that with overexpression of the *MDR1* gene, the tumor response could be only around 17%, and with no overexpression of the *MDR1* gene, the tumor response could reach 68% [17].

In a meta-analysis of 31 studies, Trock et al. concluded that chemoresistance occurs three times more often in tumors overexpressing P-gp compared to tumors with no P-gp expression. Therefore, chemoresistance is a determinant factor in tumor response to a particular chemotherapy regimen [18].

**Fig. 3:**
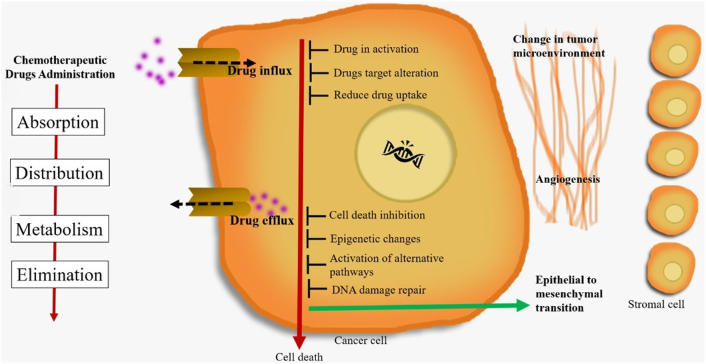
Mechanisms of drug resistance in cancer (Adapted from Housman et al. [19] and Holohan et al. [20]).

Housman et al. (2014) proposed factors influencing chemoresistance include drug inactivation, changes in drug targets, overexpression of ABC transporters, epithelial-to-mesenchymal transition, apoptotic dysregulation, and cancer stem cells, as shown in Fig. 3 [19].

## 4 Measurement of chemotherapy response

Neoadjuvant chemotherapy has traditionally been used to treat locally advanced and initially inoperable breast cancer. One of the main reasons for applying a systemic therapy before rather than after curative surgery is the potential size reduction of a malignant tumor, which is thought to permit less-invasive curative surgery. In addition, clinical and pathological remission can be achieved before surgery, which can improve outcomes. Unfortunately, although in many cases a clinically meaningful remission can be achieved, not all patients benefit equally. Some tumors even increase in size despite ongoing chemotherapy, suggesting resistance. Nevertheless, neoadjuvant and adjuvant chemotherapy are still applied empirically, since no clinical tests currently exist that would allow reliably predicting the response to and benefit from a particular chemotherapy. Therefore, the effectiveness of the chemotherapy response must be assessed.

**Fig. 4:**
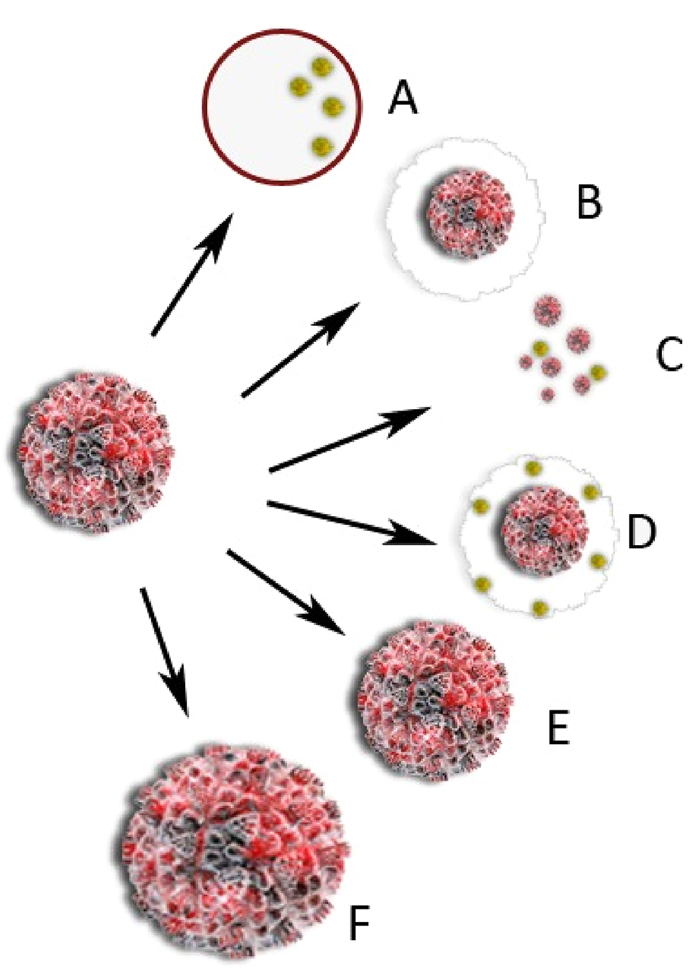
Tumor shrinkage patterns after neoadjuvant chemotherapy. (A) Pathological complete response [i.e., no residual tumor or only residual ductal carcinoma in situ (DCIS), defined as ypT0/is], (B) concentric shrinkage (i.e., only one residual invasive tumor foci, without DCIS), (C) multifocal shrinkage (i.e., more than 2 invasive tumor foci, with/without DCIS), (D) diffuse shrinkage (i.e., a main residual invasive (PD). Type 1 shrinkage is shown by (A, B). Type 2 shrinkage is represented by (C–F) [23].

Surgical planning may be affected by tumor response patterns. Concentric shrinkage or a scattergun or honeycomb response may occur (Fig. 4); in the latter, the remaining carcinoma appears as many scattered foci over an ill-defined tumor bed [21]. This pattern of reaction is especially challenging when planning surgical treatments since clear margins are more difficult to attain [22].

The effectiveness of chemotherapy can be assessed clinically or pathologically. Clinical changes from chemotherapy are measured, such as tumor shrinking, which is an indicator of a good response [24,25]. This could be evaluated after chemotherapy by physical examination, mammography, ultrasonography, computed tomography (CT) scan, or magnetic resonance imaging (MRI) [26] after at least 2–3 administrations of chemotherapy [25,27]. The accuracy for determining pCR in locally advanced breast cancer after neoadjuvant chemotherapy is 57% for physical exam, 74% for mammography, 79% for ultrasonography, and 93% for MRI [28].

Each measurement method has its advantages and disadvantages. Clinical measurement with a caliper is very easy to do but has drawbacks. Sometimes discrepancy exists between the chemotherapy response assessed by clinical examination and pathological study of the surgical specimen.

The presence of solid fibroglandular tissue and posttherapy fibrosis can cause the amount of residual illness to be overestimated during physical examination. Mammography and ultrasound had 74% and 79% accuracy for detecting postneoadjuvant pathologic tumor response, respectively, in a report of six investigations [28]. Mammography has been demonstrated to be more sensitive than physical examination in detecting the presence of residual tumor following treatment, although it is less specific and may underestimate the degree of treatment response [29,30]. After neoadjuvant therapy, ultrasound has proven a better predictor of pathologic tumor size than mammography [28,31]. In addition, when compared to mammography and physical examination, ultrasound is the most accurate predictor of axillary lymph node response [32]. When both modalities are negative, the combination of mammography and ultrasound appears to be the best technique for predicting complete pathologic response (80% chance) [31,33].

Breast MRI is the most sensitive imaging modality for detecting breast cancer [30,34] and the most reliable imaging modality for assessing tumor response to neoadjuvant therapy [28,35,36]. The positive predictive value (ability to accurately identify the presence of residual disease at the final pathologic examination) was high in a combined analysis of six investigations, at 93 [28]. The negative predictive value (ability to accurately identify the absence of disease at the final pathologic examination) was only moderate (65%), lowering the overall diagnostic accuracy to 84% [28,30].

One study divided MRI-based response patterns of breast carcinomas before and after neoadjuvant chemotherapy into six types based on Kim et al.’s classification: type 0 (complete radiologic response), type 1 (concentric shrinkage >3 mm without surrounding lesions), type 2 (crumbling: shrinkage with residual multi-nodular lesions), type 3 (diffuse contrast enhancement in entire quadrants), type 4 (stable disease, i.e., no response, shrinkage 3 mm or increase 3 mm), type 5 (progressive disease, i.e., increase in tumor size >3 mm or new lesion). The largest diameter of the largest breast lesion was measured in one image on a T1-weighted MRI sequence at peak enhancement, i.e., first dynamic phase after contrast injection. Multiplanar reconstructions were used by the assessors to determine the maximum tumor diameter [37].

The residual tumor size measured on MRI and the pathologic tumor size determined following surgical excision are generally in good agreement. A systematic review by Lobbes et al. discovered that MRI can overestimate or underestimate the residual tumor size, with a median correlation coefficient of 0.70 (range, 0.21–0.98) [35].

No criteria currently exist for reporting the tumor response to neoadjuvant therapy based on imaging. The current edition of the American College of Radiology Breast Imaging Reporting and Data System lacks clear guidelines on how to submit follow-up imaging for assessing the response to therapy. Typically, the biggest dimension measurement is used to compare tumor size before and after treatment. Descriptive patterns of tumor response, such as mammographic lesion density decrease, change in internal echotexture, and concentric versus fragmented lesion shrinking with intervening normal-appearing tissue, may also be beneficial [30].

In clinical practice, fluorodeoxyglucose positron emission tomography (FDG PET) imaging is the most commonly used molecular imaging agent for imaging tumor glycolytic metabolism with PET. FDG PET imaging can be used for optional systemic staging and restaging of patients with stage III illness, locally progressed and inflammatory breast cancer, and recurrent and/or metastatic breast cancer, according to the most recent National Comprehensive Cancer Network guidelines. It is especially useful when the results of standard staging investigations (CT or MRI with bone scan) are inconclusive [25].

FDG PET has been evaluated in many studies for predicting the pathologic response to neoadjuvant treatment [[38], [39], [40], [41], [42]]. The largest prospective multicenter analysis included 272 examinations of 104 patients with newly diagnosed large or locally advanced non-inflammatory primary breast cancer who were also enrolled in a trial comparing two preparatory chemotherapy regimens [43]. A threshold of a 45% drop in standardized uptake value accurately identified 11 of 15 histopathologic responders following the first cycle of chemotherapy. Nonresponders had a negative predictive value of approximately 90% (34 of 38). FDG PET imaging thus appears to help predict neoadjuvant chemotherapy response and detect early nonresponders [30].

The effectiveness of chemotherapy on cancer cells is measured in terms of response. In 1981, the World Health Organization (WHO) developed a method for assessing tumor response bi-dimensionally, measuring the longest size of a tumor and its size perpendicular to that length [24]. In 1999, a new tumor response assessment method known as the Response Evaluation Criteria in Solid Tumors (RECIST) was introduced to measure uni-dimensional tumors. It was updated in 2009 (RECIST 1.1). The RECIST and WHO criteria each have advantages and disadvantages. Currently, RECIST is most used because the criteria are simpler [26,44,45].

Currently, in addition to RECIST 1.1, the Positron Emission Tomography Response Criteria in Solid Tumors (PERCIST) is available [30,46]. The response to therapy is measured as a continuous variable and expressed as a percentage difference in the SUL peak (or sum of lesion SULs) between pre- and posttreatment scans. In simple terms, a full metabolic response is the visible removal of all metabolically active tumor cells. A partial response is defined as a decrease in SUL peak of more than 30% and 0.8 units between the most intense lesion before therapy and the most intense lesion after treatment, which does not have to be the same lesion. Progressive illness is defined as an increase in SUL peak of more than 30% and new lesions of more than 0.8 units, if verified. Another indicator of advancement is a 75% rise in total lesion glycolysis [46].

Based on RECIST, responses are classified as a complete response, partial response, stable disease, or progressive disease. Complete response is the loss of all tumor masses. Partial response is a tumor that becomes at least 30% smaller than the longest diameter of the tumor. Stable disease is where the tumor size decrease is not enough for a partial response, but the tumor does not increase in size and become a progressive disease. Progressive disease is where a tumor increases by at least 20% of the longest diameter [24,45].

Pathological responses are more meaningful and reliable markers of life expectancy than clinical responses. However, pathological evaluation is more difficult because histopathological tissue assessment is conducted using core biopsy or during surgery. Pathological responses after chemotherapy induction in breast cancer are predictors of DFS and overall survival. Several classifications have been recommended, such as the Miller and Payne classification (Table 4), which is based on cell loss after more reliable therapy [[47], [48], [49]]. Based on this classification, pathological responses are divided into five levels based on the degree of death and cell damage [49].

**Table 4:** Classification of cancer cell changes, based on Miller and Payne [49].

Grade	Change in cellularity
1	There are no changes in cancer cells. There is no change in overall cells.
2	Minor cancer cell lost. Overall cells lost <30%.
3	Cancer cell reduction is estimated at 30–90%.
4	Cancer cells lost.
5	Total loss of invasive cancer cells. No cancer cell is identified. Only fibroelastic stroma remains. Ductal carcinoma in situ could persist.

In the Miller–Payne system, pathologic response is divided into five grades based on comparison of tumor cellularity between pre–neoadjuvant therapy core biopsy specimens and definitive surgical specimens. A grade of 1 indicates no change or some alteration in individual malignant cells but no reduction in overall cellularity (pathologic nonresponse); a grade of 2, minor loss of tumor cells but still high overall cellularity of up to 30% (pathologic partial response); a grade of 3, an estimated reduction in tumor cells of between 30% and 90% (pathologic partial response); a grade of 4, a marked disappearance of tumor cells such that only small clusters or widely dispersed individual cells remain, with more than 90% loss of tumor cells (almost pathologic complete response); and a grade of 5, no malignant cells identifiable in slices from the site of the tumor and only vascular fibroelastic stroma remaining, often containing macrophages, but ductal carcinoma in situ may be present (pathologic complete response) [49].

Clinical responses are often inconsistent with pathological responses, especially since the first stage of DNA fragmentation is difficult to evaluate with certainty. Therefore, the evaluation of specimens obtained from mastectomy is the gold standard for determining response to therapy [50].

## 5 Suspected chemoresistance

Chemoresistance is suspected if tumor cells show no adequate changes both subjectively and objectively after chemotherapy. Subjective symptoms indicate the effectiveness of chemotherapy, such as feeling better, feeling “fresher,” increased Karnofsky score, decrease or disappearance of tumor smell, decreased pain, and decreased cough. Objective symptoms are measured by physical examination using pathological or tumor markers. In general, measuring the response after at least 2–3 cycles of chemotherapy is essential because one cycle is inadequate to evaluate the effectiveness [24].

## 6 Influencing risk factors of chemoresistance

Several risk factors affect the probability of chemoresistance. In studies, these factors potentially cause treatment bias. Age, subtype, and stage of cancer progression are important patient factors that can cause treatment bias.

Age-related changes can also affect pharmacodynamics, often resulting in increased resistance to the anti-tumor activity of chemotherapeutic drugs. Elderly people are more likely to express the multidrug resistance gene, which causes tumor cells to extrude natural drugs such as antibiotics and plant derivatives.

This mechanism may account for the drug resistance often seen in older patients with acute myeloid leukemia. In addition, chemotherapeutic drugs that depend on inducing apoptosis are less effective if a significant proportion of tumor cells have lost this capacity [51].

The tumoricidal effects of chemotherapy and radiotherapy are greatest in well-oxygenated cells that are rapidly proliferating. Therefore, treatment in older patients may be less effective, because tumors in this age group are often relatively anoxic (impaired circulation) and indolent (due to a natural-selection process because more-aggressive tumors typically cause death at an earlier age) [51].

Breast cancer is classified into molecular subtypes. Among these, triple-negative breast cancer is considered one of the most aggressive, and its chemotherapy response rate is higher compared to the others. Despite the aggressive nature of triple-negative breast cancer, 20% of patients present a pathologic complete response (pCR) after neoadjuvant chemotherapy. However, triple-negative breast cancer patients who do not achieve pCR are several times more likely to experience an early recurrence and die from metastatic disease compared to other subtypes. On the whole, triple-negative breast cancer patients have a significantly worse overall survival compared to those with other subtype breast tumors, despite better pCR rates, a phenomenon termed the “triple-negative paradox.” The differences in clinical outcomes following neoadjuvant treatment imply that a subset of triple-negative breast cancers are sensitive to chemotherapy, whereas the majority become resistant during treatment or are intrinsically less susceptible. Both mechanisms are likely present in these tumors [52].

## 7 Predictors of chemotherapy response

At present, no indicators can reliably predict breast cancer's chemotherapy responses. However, some biomarkers are frequently used to predict these.

## 8 Tumor type and grading

The type and grading of tumors are related to chemotherapy responses. Invasive lobular carcinoma responds less favorably to chemotherapy compared to invasive ductal carcinoma [53]. Tumor grading in relation to chemotherapy response has also been studied. Although grade III tumors are more responsive to chemotherapy, many studies have found no meaningful relationship in predicting the response to chemotherapy yet [44,54].

## 9 Hormonal receptors: estrogen receptors and progesterone receptors

Several studies found that cells that do not express ER or PR respond better to chemotherapy than those that express ER/PR [44,55,56].

## 10 Human epidermal growth factor receptor 2

HER-2 plays an important role in the growth of breast cancer. Overexpression of HER-2 is found in 25% of breast cancers. Tumors with HER-2 expression are more aggressive. Some studies suggest that overexpression of HER-2 in breast cancer is associated with responses to cyclophosphamide, methotrexate, and 5-fluorouracil chemotherapy and anthracycline-based regimens [44].

## 11 Tumor proliferation: the Ki-67 protein

Breast cancer tumors with high cell proliferation and expressing Ki-67 have a good response to chemotherapy. Changes in Ki-67 expression are associated with an increased response to chemotherapy [57,58]. Although these findings have been documented worldwide in many studies, no general agreement exists about them [59].

## 12 Gene apoptosis: tumor protein p53 and B-cell lymphoma 2

Many chemotherapy agents kill cancer cells by inducing apoptosis. Therefore, proteins related to apoptotic pathways (p53 and BCL-2) have been studied to predict the response to chemotherapy [60]. The p53 gene is the most frequently mutated in humans. This mutation occurs in approximately 50% of cancers in humans [61,62]. The p53 protein is involved in the apoptotic pathway by inducing cell cycle termination and initiating apoptosis. However, the use of p53 as a predictor of chemotherapy response is still disappointing due to other factors, such as the type of tissue, use of chemotherapy drugs, and status of tumor mutation. The *BCL-2* gene also plays a role in inhibiting apoptosis. BCL-2 is one of the proteins involved in apoptosis. However, in a clinical context, the relationship between BCL-2 and Bax protein as predictors of chemotherapy response is still unclear [44].

In clinical setting Favor Adjuvant Chemotherapy if; ER Negative, Ductal Histology, Grade 3, High proliferation, High uPA and PA1, Basal and HER2 positive, High Mammaprint or OncotypeDx.

## 13 New methods for predicting chemotherapy response

Resistance is frequently diagnosed during treatment after several cycles of drug administration. Some methods for predicting drug resistance are still being studied, such as chemoresistance and chemosensitivity assays, multigene expressions, and PET assays. These methods are based on evaluating the molecular processes and metabolic activity of cancer cells [11].

## 14 Chemotherapy assay

Various in vitro chemoresistance tests and chemosensitivity assays have been developed to provide information about the characteristics of malignancies in individual patients and predict the potential cancer response to a certain drug. These tests are used to choose a treatment regimen for each patient. Several tests differ in the processing of biological samples and detection methods. However, they involve the same principle and protocol components, such as (1) cell isolation and in vitro media formation, (2) cell incubation with various drugs, (3) cell survival assessment, and (4) interpretation of results. These are related to the recommendations of the National Comprehensive Cancer Network, American Society of Clinical Oncology, and European Society for Medical Oncology 2018. They have only been approved for use in research on chemotherapy assays such as The Extreme Drug Resistance assay (EDR® assay), Histoculture Drug Resistance Assay (HDRA®), and ChemoFX Assay (Precision Therapeutics) [63].

## 15 Multigene biomarkers

The development of DNA microarrays and proteomics technology is used to gain a better understanding of genes that may be involved in regulating tumor cell responses to chemotherapy. The technology has led to the identification of biomarker panel expressions that could predict chemotherapy responses and help choose the most appropriate chemotherapy regimen for certain molecular characteristics [64]. Gene expression profiles from tumor samples produce markers to detect certain cancer features, such as metastasis and chemoresistance. These markers contain genes whose expression changes in some types of indicated cancer [59]. MammaPrint is a breast cancer gene marker (70-gen) that could be used in metastasis assessment. OncotypeDX (Genomic Health Inc., Redwood City, CA, USA) is also a marker of breast cancer genes (21-gen) that could be used in chemoresistance assessment [64]. The taxane-based chemotherapy signature (TAXSig) is used in predicting taxane-based chemotherapy responses in breast cancer patients. However, not enough data exists for global recommendation [65].

## 16 PET assay

PET has been used clinically to detect cancer localization. Recently, this method has been applied to determine the metabolic activity of cancerous tissues. PET is also used in vitro for testing cancer cell cultures by measuring the metabolic activity of cancer cells [66].

## 17 Use of chemotherapy predictors

**Fig. 5:**
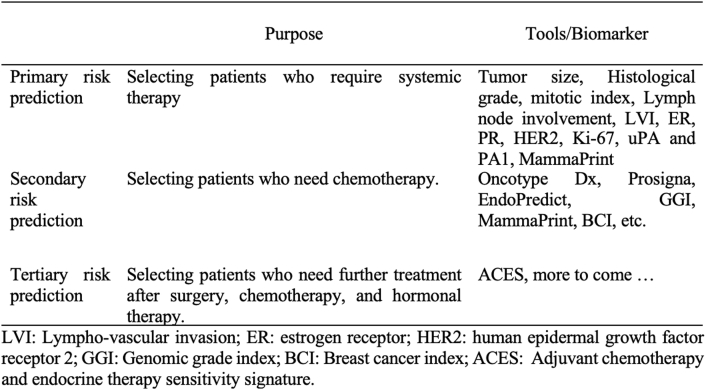
Conceptual framework for risk stratification and currently available prognostic and predictive tools (Adapted from Győrffy et al. [64]).

Using parameters as predictors of chemotherapy response involves three levels [64]. The first level is primary risk prediction (tumor size, nodal status, grade, ER, HER-2, adjuvant online, MammaPrint), which is used to identify patients who can have surgical therapy only without systemic chemotherapy. The second level is secondary risk prediction (Oncotype Dx, Prosigna, EndoPredict/EPclin, GGI, MammaPrint, breast cancer index, etc.), which is used by patients with hormonal positive receptors who can be treated surgically and hormonally without systemic chemotherapy. The third level is tertiary risk prediction such as adjuvant chemotherapy and endocrine therapy sensitivity signature (ACES, etc.), which is used in patients who cannot have therapy with surgery, hormonal therapy, and systemic chemotherapy, so they are candidates for clinical trials or new drug development (Fig. 5) [64].

## 18 Prevention of chemoresistance

### 18.1 Selection of appropriate chemotherapy regimens

Selection of chemotherapy regimens is essential in preventing resistance to a chemotherapy drug. Regimen selection has several parameters, including type, grade, subtype, ER, PR, Her2, Ki-67, and gene expression [25]. Many types of software can help in the selection of chemotherapy regimens and predict responses, including MD Anderson chemotherapy calculators, the SVM (Support Vector Machine) Chemotherapy Response Support Calculator, and the Breast Cancer Treatment Outcome Calculator [[67], [68], [69]].

### 18.2 Combination of chemotherapy

One of the most effective ways to prevent chemoresistance is by using a combination of chemotherapy. Combined chemotherapy uses drugs that work with different mechanisms, resulting in reduced chances of resistant cancer cells. When different effects of drugs are combined, each drug is used at optimal doses without unbearable side effects. As well as combinations with other chemotherapy drugs, chemotherapy treatment could be combined with targeting therapy, radiotherapy, or other therapeutic modalities [70].

### 18.3 Monitoring and evaluation of chemotherapy

Monitoring and collecting chemotherapy data are important for evaluating the implementation of chemotherapy. Continuous evaluation of clinical response data and chemotherapy regimens is done according to the characteristics of patients and tumors [25].

### 18.4 Prevention of emergent multidrug resistance

Many studies have developed new drugs to prevent multidrug, including the following [12].

(1) Drug excretion increases with excessive expression of ABC transporters during chemotherapy. Curcuminoids and NSC77037 reduce NF-κB transcription, and PSC833 and BVDU suppress amplification of the *ABCB1* gene. In addition, VX710, XR9576, PSC833, NSC23925, bexarotene, NS-398, dexrazoxane, curcuminoid, and P85 directly inhibit the expression of P-gp.
(2) Anticancer drugs conjugated to glutathione (GSH) result in inactivation of drugs. This is catalyzed by glutathione s-transferase (GST) through the utilization of energy derived from ATP hydrolysis. Ethacrynic acid, P85, and selenium compounds inhibit GST activity, and selenium compounds even reduce GSH levels.
(3) Apoptosis starts through two pathways: the intrinsic pathway (mediated by mitochondria) and the extrinsic pathway. NSC23925 inhibits the expression of Bcl-xL, an anti-apoptotic protein, and results in promoting apoptosis.

Most of these drugs are still under trial.

## 19 Conclusion

Chemotherapy is an essential treatment in the management of breast cancer. At present, breast cancer shows good responses to chemotherapy. Chemotherapy is carried out based on the selection of regimens for the specific individual and tumor characteristics, combination therapy, continuous monitoring, and evaluation and use of drugs to prevent emergent chemoresistance.

## Provenance and peer review

Not commissioned, externally peer-reviewed.

## Funding

No funding or sponsorship.

## Ethical approval

Review article applicable for exemption by our Institutional review board.

## Consent

This manuscript does not involve human participants, human data, or human tissue.

## Author contribution

Prihantono: Design, editing and writing of the manuscript, supervision of the paper, and approved the final manuscript.

Muhammad Faruk: Editing, final review and approved the final manuscript.

## Guarantor

Prihantono.

## Declaration of competing interest

The authors declare that they have no conflict of interests.
